# The *Helicobacter pylori* Heat-Shock Repressor HspR: Definition of Its Direct Regulon and Characterization of the Cooperative DNA-Binding Mechanism on Its Own Promoter

**DOI:** 10.3389/fmicb.2018.01887

**Published:** 2018-08-14

**Authors:** Simona Pepe, Eva Pinatel, Elisabetta Fiore, Simone Puccio, Clelia Peano, Tarcisio Brignoli, Andrea Vannini, Alberto Danielli, Vincenzo Scarlato, Davide Roncarati

**Affiliations:** ^1^Department of Pharmacy and Biotechnology (FaBiT), University of Bologna, Bologna, Italy; ^2^Institute of Biomedical Technologies, National Research Council, Milan, Italy; ^3^Humanitas Clinical and Research Center, Milan, Italy; ^4^Institute of Genetic and Biomedical Research, National Research Council, Milan, Italy

**Keywords:** heat-shock response, HspR repressor, ChIP-seq, RNA-sequencing, transcriptome

## Abstract

The ability of pathogens to perceive environmental conditions and modulate gene expression accordingly is a crucial feature for bacterial survival. In this respect, the heat-shock response, a universal cellular response, allows cells to adapt to hostile environmental conditions and to survive during stress. In the major human pathogen *Helicobacter pylori* the expression of chaperone-encoding operons is under control of two auto-regulated transcriptional repressors, HrcA and HspR, with the latter acting as the master regulator of the regulatory circuit. To further characterize the HspR regulon in *H. pylori*, we used global transcriptome analysis (RNA-sequencing) in combination with Chromatin Immunoprecipitation coupled with deep sequencing (ChIP-sequencing) of HspR genomic binding sites. Intriguingly, these analyses showed that HspR is involved in the regulation of different crucial cellular functions through a limited number of genomic binding sites. Moreover, we further characterized HspR-DNA interactions through hydroxyl-radical footprinting assays. This analysis in combination with a nucleotide sequence alignment of HspR binding sites, revealed a peculiar pattern of DNA protection and highlighted sequence conservation with the HAIR motif (an HspR-associated inverted repeat of *Streptomyces* spp.). Site-directed mutagenesis demonstrated that the HAIR motif is fundamental for HspR binding and that additional nucleotide determinants flanking the HAIR motif are required for complete binding of HspR to its operator sequence spanning over 70 bp of DNA. This finding is compatible with a model in which possibly a dimer of HspR recognizes the HAIR motif overlapping its promoter for binding and in turn cooperatively recruits two additional dimers on both sides of the HAIR motif.

## Introduction

*Helicobacter pylori* represents one of the most widespread human pathogens, recognized as the principal causative agent of different gastrointestinal severe diseases such as atrophic gastritis, peptic ulcer, MALT-lymphoma and gastric adenocarcinoma (Gisbert and Calvet, 2011; Salama et al., 2013). A peculiar feature of *H. pylori* is its small genome (1.66 Mb), characterized by a relative low abundance of genes encoding regulators of transcription. In this respect, it has been speculated that this paucity of transcriptional regulators could reflect the adaptation of *H. pylori* to its very restricted niche in the mucus layer of the human stomach and could be linked to the lack of competition from other microorganisms (Marshall et al., 1998). To date, only 17 transcriptional regulators have been identified and shown to control key physiological responses of the bacterium, which are necessary to successfully colonize the gastric niche (Scarlato et al., 2001).

*Helicobacter pylori* transcriptional regulators appear to be arranged in different regulatory modules, transducing separate environmental inputs. Such regulatory modules are constituted by a master regulator, followed by intermediate regulators and regulatory interactions finally resulting into a coordinated output of target gene’s expressions (Danielli et al., 2010). Intriguingly, these regulatory modules do not appear as segregated, but highly interconnected and show a significant crosstalk among different signal transduction pathways. *H. pylori* does not possess a homologue of the *Escherichia coli* heat-shock sigma factor σ^32^. However, the heat stress response is governed by two dedicated transcriptional repressors, HrcA and HspR that negatively regulate the expression of the highly conserved class of stress-induced proteins, known as Heat-Shock Proteins (HSPs). The main function of HSPs is to assist the folding of newly synthetized polypeptides, as well as assembly, transport and degradation of cellular proteins under both normal and adverse growth condition (Roncarati and Scarlato, 2017). Besides their general role in protecting cellular proteins against different kind of stresses and in maintaining cellular homeostasis, some HSPs and chaperones are considered virulence factors and seem to be involved in specific pathogenic processes. Specifically, several lines of evidence show that the heat-shock proteins of *H. pylori* play also non-canonical roles and some of them seem to have undertaken additional functions during the interaction with the host (Evans et al., 1992; Huesca et al., 1996; Phadnis et al., 1996; Dunn et al., 1997; Kao et al., 2016). Because of these important functions in the cell, *H. pylori* has developed regulatory strategies to tightly modulate HSPs expression level in response to environmental signals.

In previous works (Spohn and Scarlato, 1999; Spohn et al., 2004) it has been demonstrated that the transcription of *groES-groEL*, *hrcA-grpE-dnaK*, and *cbpA-hspR-rarA* operons, encoding the major chaperones of *H. pylori*, is negatively regulated by HspR and/or HrcA repressors, with the former acting as the master regulator of this circuit. Indeed, HspR represses transcription of the *cbpA* operon alone, thereby auto-regulating its own synthesis, while it represses the expression of the other two heat-shock operons *groES-groEL* and *hrcA-grpE-dnaK* in combination with HrcA. Genes with sequence similarity to *H. pylori* *hspR* were annotated in several other bacteria, including species belonging to the *Streptomyces* genus. In particular, *H. pylori* HspR is a homologue of the repressor that controls the expression of the *dnaK* operon in *Streptomyces coelicolor* by binding three copies of the HspR consensus binding sequence called HAIR (for HspR Associated Inverted Repeat) (Bucca et al., 1995; Grandvalet et al., 1999). Also in *Streptomyces albus* it was demonstrated that HspR binds to an inverted repeat identical to *S. coelicolor* HAIR sequence, mapping in the promoter region of the protease gene *clpB* (Grandvalet et al., 1999). In *H. pylori*, DNaseI footprinting experiments with the purified protein showed that HspR binds extended DNA regions located in the promoters of the three heat-shock operons and recognizes sequences similar to the HAIR motif (Delany et al., 2002c; Roncarati et al., 2007). While in the case of *cbpA* promoter HspR binding occurs in close proximity to the transcription start site, on the *groEL* and *hrcA* promoters HspR binds far upstream of the core promoter region, in an atypical position for a repressor (Roncarati et al., 2007). The identification of additional genes controlled by HspR was pursued through array-based whole transcriptome analyses both in *H. pylori* (Roncarati et al., 2007) and in several other bacterial species (Stewart et al., 2002; Andersen et al., 2005; Schmid et al., 2005; Holmes et al., 2010). Moreover, from an *in vitro* selection of genomic DNA fragments bound by purified *H. pylori* HspR protein, two novel HspR binding sites were identified in the 3′ regions of both *speA* and *tlpB* genes coding for proteins with functions unrelated with those of chaperones (Delany et al., 2002c).

In the present study, by combining ChIP-sequencing and RNA-sequencing we investigated more in detail the direct or indirect contribution of *H. pylori* HspR to the heat-shock regulon. While the heat-shock regulon includes many genes with key cellular functions, HspR only binds to a limited number of genomic sites. Furthermore, by means of the high-resolution hydroxyl-radical footprinting technique, we further characterized the HspR-DNA interactions at the molecular level. The data provide a more detailed comprehension of the interaction between HspR and its target DNA sequences and, at least on its own promoter, let us to propose a cooperative DNA-binding mechanism of three HspR dimers per operator sequence.

## Materials and Methods

### Bacterial Strains and Growth Conditions

*Helicobacter pylori* strains (**Table 1**) were recovered from frozen glycerol stocks on Brucella broth agar plates, containing 5% fetal calf serum (FCS), in a 9% CO_2_-91% air atmosphere at 37°C and 95% humidity in a water jacketed incubator (Thermo Scientific). Liquid cultures were grown in Brucella Broth supplemented with 5% FCS with gentle agitation (120 rpm). *E. coli* strains DH5α and BL21 (DE3) (**Table 1**) were grown on Luria-Bertani (LB) agar plates or LB liquid broth with vigorous agitation (250 rpm); when required, ampicillin was added to the medium to achieve a final concentration of 100 μg/ml.

**Table 1:** Bacterial strains and plasmids used in this study.

Bacterial strains/ Plasmids	Description	Source/ Reference
***Strains***		
*H. pylori* G27 wild type	Clinical isolate, wild type	Xiang et al., 1995
*H. pylori* G27 (*hspR::Km*)	G27 derivative; bp 66 to 334 of the *hspR* coding sequence replaced by a Kanamycin (*Km*) cassette.	Spohn and Scarlato, 1999
*H. pylori* G27 (*vacA::Pcbpwt-lux*)	G27 derivative; containing the P*cbp* wild type promoter region upstream of the *luxC* gene in the *vacA* locus; Cp^r^	This work
*H. pylori* G27 (*vacA::Pcbpwt-lux*, *hspR::Km*)	G27 derivative; containing the P*cbp* wild type promoter region upstream of the *luxC* gene in the *vacA* locus and the *hspR* coding sequence (bp 66 to 334) replaced by a Kanamycin (*Km*) cassette; Cp^r^, Km^r^	This work
*H. pylori* G27 (*vacA::PcbpM1 + 2-lux*, *hspR::Km*)	G27 derivative; containing the P*cbp* HAIR-mutant promoter region upstream of the *luxC* gene in the *vacA* locus and the *hspR* coding sequence (bp 66 to 334) replaced by a Kanamycin (*Km*) cassette; Cp^r^, Km^r^	This work
*H. pylori* G27 (*vacA::PcbpM1 + 2-lux*)	G27 derivative; containing the P*cbp* HAIR-mutant promoter region upstream of the *luxC* gene in the *vacA* locus; Cp^r^	This work
*E. coli* DH5α	*supE44 ΔlacU169 (φ80 lacZΔM15) hsdR17 recA1 endA1 gyrA96 thi-1 relA1*	Hanahan, 1983
*E. coli* BL21(DE3)	*hsdS gal (λcIts857 ind1 Sam7 nin5 lacUV5-T7 gene 1).*	Studier et al., 1990
***Plasmids***		
pGEM-T-Easy	Cloning vector, Amp^r^	Promega
pGEM-T-Easy-RBS gro	pGEM-T-Easy derivative, containing a 147 bp DNA fragment corresponding to the region from 9,452–9,543 of *H. pylori* G27 genome amplified by PCR with oligonucleotides RBS GroF/RBS GroR. This region corresponds to a portion of the promoter region of HPG27_RS00075 (HP0011 according to 26695 annotation).	This work
pGEM-T-Easy-RBS hrc	pGEM-T-Easy derivative, containing a 252 bp DNA fragment corresponding to the region from 118,944–119,035 of *H. pylori* G27 genome amplified by PCR with oligonucleotides RBShrcF/PhrcF. This region corresponds to a portion of the promoter region of HPG27_RS00580 (HP0111 according to 26695 annotation).	This work
pGEM-T-Easy-HBS cbp	pGEM-T-Easy derivative, containing a 146 bp DNA fragment corresponding to the region from 433,049 to 433,140 of *H. pylori* G27 genome amplified by PCR with oligonucleotides HBSCbpF/HBSCbpF. This region corresponds to a portion of the promoter region and coding sequence of HPG27_RS02130 (HP1024 according to 26695 annotation).	This work
pGEM-T-Easy-HBSspeA	pGEM-T-Easy derivative, containing a 135 bp DNA fragment corresponding to the region from 1,035,104–1,035,195 of *H. pylori* G27 genome amplified by PCR with oligonucleotides HBSspeAF /HBSspeAR. This region corresponds to a portion of the coding sequence of HPG27_RS02130 (HP0422 according to 26695 annotation).	This work
pGEM-T-Easy-HBScbpM1	pGEM-T-Easy derivative, containing a 91 bp DNA fragment corresponding to the region from 432,991–433,071 of *H. pylori* G27 genome generated annealing oligonucleotides Mut1F/Mut1R. This region corresponds to a portion of the promoter region and coding sequence of HPG27_RS02130 (HP1024 according to 26695 annotation).	This work
pGEM-T-Easy-HBScbpM2	pGEM-T-Easy derivative, containing a 146 bp DNA fragment corresponding to the region from 433,049–433,140 of *H. pylori* G27 genome amplified by all around PCR with oligonucleotides Mut2F/Mut2R and using as DNA template the plasmid pGEM-T-Easy-HBScbp. This region corresponds to a portion of the promoter region and coding sequence of HPG27_RS02130 (HP1024 according to 26695 annotation).	This work
pGEM-T-Easy-HBScbpM1 + 2	pGEM-T-Easy derivative, containing a 146 bp DNA fragment corresponding to the region from 433,049–433,140 of *H. pylori* G27 genome amplified by all around PCR with oligonucleotides Mut2F/Mut2DR and using as DNA template the plasmid pGEM-T-Easy-HBScbp. This region corresponds to a portion of the promoter region and coding sequence of HPG27_RS02130 (HP1024 according to 26695 annotation).	This work
pGEM-T-Easy-HBScbpM3	pGEM-T-Easy derivative, containing a 91 bp DNA fragment corresponding to the region from 432,991–433,071 of *H. pylori* G27 genome generated annealing oligonucleotides Mut3F/Mut3R. This region corresponds to a portion of the promoter region and coding sequence of HPG27_RS02130 (HP1024 according to 26695 annotation).	This work
pGEM-T-Easy-HBScbpM4	pGEM-T-Easy derivative, containing a 91 bp DNA fragment corresponding to the region from 432,991–433,071 of *H. pylori* G27 genome generated annealing oligonucleotides Mut4F/Mut4R. This region corresponds to a portion of the promoter region and coding sequence of HPG27_RS02130 (HP1024 according to 26695 annotation).	This work
pGEM-T-Easy-HBScbpM5	pGEM-T-Easy derivative, containing a 91 bp DNA fragment corresponding to the region from 432,991–433,071 of *H. pylori* G27 genome generated annealing oligonucleotides Mut5F/Mut5R. This region corresponds to a portion of the promoter region and coding sequence of HPG27_RS02130 (HP1024 according to 26695 annotation).	This work
pVCC	Vector carrying the *luxCDABE* cassette	Vannini et al., 2014
pVAC::Km	Cloning Vector, Km^r^	Delany et al., 2002b
pVAC::CAT	pVAC::Km derivative, carrying a BglII/BamHI *cat* cassette from pBS::*cat* (Vannini et al., 2012).	This work
pVAC-Pcbpwt-lux	pVAC-CAT derivative, containing a 146 bp DNA fragment amplified by PCR with oligonucleotides HBSCbpFEco/HBSCbpRBamHI, encompassing the P*cbp* wt promoter region and a 1,000 bp DNA fragment amplified by PCR with oligonucleotides LuxF/LuxR of the *luxC* gene.	This work
pVAC-PcbpM1+2-lux	pVAC-CAT derivative, containing a 146 bp DNA fragment amplified by PCR with oligonucleotides HBSCbpFEco/HBSCbpRBamHI (using as DNA template the plasmid pGEM-T-Easy-HBScbpM1 + 2), encompassing the P*cbp* M1 + 2 promoter region and a 1,000 bp DNA fragment amplified by PCR with oligonucleotides LuxF/LuxR of the *luxC* gene.	This work
pGEM3-*hspR::Km*	pGEM3 vector carrying the *Campylobacter coli* Kanamycin cassette flanked by a 1067 bp fragment comprising the *cbpA* gene (HPG27_RS02130) and a 716 bp fragment comprising the 5′ region of the *rarA* gene (HPG27_RS02120).	Spohn and Scarlato, 1999
pET22b	Expression vector, allow C-terminal histidine-tag gene fusion; Amp^r^	Novagen
pET22b-HspR	pET22b derivative, containing the HspR coding sequence amplified by PCR	Spohn and Scarlato, 1999

### RNA Isolation

*Helicobacter pylori* strains (**Table 1**) were grown with gentle agitation (120 rpm) in 30 ml of Brucella broth at 37°C until mid-exponential phase (OD = 0.7). For heat-shock treatment, the wild type (WT) culture was split into 15 ml-aliquots and one sample was subjected to heat-shock at 42°C for 30 min (heat-shock sample, HS). A volume of 10 ml cell culture was then added to 1.25 ml of ice-cold EtOH-phenol stop solution (5% acid phenol, in EtOH) to stop growth and prevent RNA degradation. Cells were pelleted, stored at −80°C, and then used to extract total RNA with TRI-reagent (Sigma-Aldrich), according to manufacturer’s protocol.

### RNA-seq: Library Preparation, Sequencing and Analyses

Ribosomal RNAs were depleted starting from 1 μg of total RNA from each of the conditions analyzed by using the RiboZero Gram negative kit (Epicentre, Illumina). Strand specific RNA-seq libraries were prepared by using the ScriptSeq^TM^ v2 RNAseq library preparation kit (Epicentre, Illumina) starting from 50 ng of previously rRNA-depleted RNA from each biological replicate and for all the conditions analyzed. Then, each library was multiplexed in equal amounts and sequenced on a GAIIX Illumina sequencer and 85 bp reads were produced. A minimum of 7 Million reads were obtained for each of the samples and for each replica. Bowtie 2 (v2.2.6) (Langmead and Salzberg, 2012) was used to align raw reads to *H. pylori* G27 genome selecting end-to-end mapping and specifying non-deterministic option. High quality reads were selected requiring: for uniquely mapping reads MAPQ > 30 (mapping quality) and alignment score >−15; for multi-mapping reads alignment score was set ≥−15. *H. pylori* G27 RefSeq annotation (GCF_000021165.1) in the version released on sept-2017 was used as the reference for gene annotation to which we manually added validated ncRNAs (Pelliciari et al., 2017; Vannini et al., 2017) (highlighted in yellow in **Supplementary Table S1**). We also revised the annotation of protein coding genes that, based on our sequencing data, were improperly annotated as pseudogenes in this version of the reference genome (e.g., *rpoB, rpoA, hspR*), indicating them as “protein-coding^∗^” in **Supplementary Table S1**. BEDTools (v2.20.1) (Quinlan and Hall, 2010) and SAMtools (v0.1.19) (Li et al., 2009) were used to verify the library preparation and sequencing performances. In particular, we measured the level of rRNA depletion, which was very efficient (less than 6% of the mapping reads) and strand specific gene coverage, considering only strand specific reads overlapping for at least 50% of their length to the annotated transcripts (see **Supplementary Table S2**). This analysis revealed that 99% of the transcripts were covered by at least one strand specific read and a minimum of 46 reads were counted on 90% of them. The R package DESeq2 (v1.4.5) (Love et al., 2014) was then used to normalize the counts and to identify differentially expressed genes (DEGs) showing BH adjusted *p*-value (*p*adj) lower than 0.01 and log2 fold changes (log2FC) > |1| . Raw data are publicly available at Sequence Reads Archive under accession number BioProject PRJNA421261.

To evaluate functional enrichments in the DEGs lists, we retrieved COG functional classes for all the protein coding genes present in our annotation file through the NCBI CDD database (Tatusov et al., 1997). We obtained COG records for 1047 genes, 88 of them were annotated as “function unknown” or “general function prediction only” categories, so we considered a final list of 959 COG annotated genes for functional enrichment analysis. The genes classified as: (1) not coding for proteins, (2) coding for proteins but not annotated in COG or (3) annotated in COG to “function unknown” or “general function prediction only” categories were merged together into the “Unknown function” in the annotation file (see **Supplementary Table S1**).

### Chromatin Immunoprecipitation (ChIP) With α-HspR Polyclonal Antibody

Available α–HspR polyclonal antibody from immunized mice (Vannini et al., 2016) were purified by 3 sequential precipitations with 35% saturated (NH4)_2_SO_4_ and subsequent resuspension in water. *H. pylori* G27 wild type and *hspR* mutant strains were liquid-grown to an OD_600_ of 0.7, crosslinked, sonicated and immunoprecipitated as previously described (Pelliciari et al., 2017; Vannini et al., 2017). Briefly, protein-DNA complexes were chemically crosslinked with 1% of formaldehyde, and then DNA was sonicated, at high power, with Bioruptor (Diagenode). HspR-DNA complexes were immunoprecipitated by incubating whole cellular extracts with the α-HspR polyclonal antibody at a 1:30 dilution and then captured with Protein-G conjugated sepharose beads. Cross-linking was reverted for 6h at 65°C. DNA was extracted once with phenol-chloroform and further extracted with chloroform. Finally, DNA was ethanol precipitated with the addition of 1% glycogen (Sigma-Aldrich) and resuspended in 50 μl of double-distilled water as previously described (Vannini et al., 2017).

### ChIP-seq: Library Preparation, Sequencing and Analyses

Illumina libraries were prepared following the Illumina TruSeq ChIP-seq DNA sample preparation protocol starting from 5 ng of immunoprecipitated-DNA for each of the strains and each of the two biological replicates. Each library was sequenced on a GAIIx or MiSeq Illumina sequencer and 51 bp single stranded reads were produced. At least 2 Million of raw reads were obtained for each IP sample and biological replicate. Bowtie 2 (v2.2.6) (Langmead and Salzberg, 2012) was used to align raw reads deriving from Input (IP *ΔhspR)* and IP (IP *wt*) samples sequencing on the *H. pylori* G27 genome. End-to-end mapping was performed and non-deterministic option was specified. High quality reads were then selected requiring: for uniquely mapping reads MAPQ > 30 (mapping quality) and alignment score > −10 while, for multi-mapping reads, the alignment score was set ≥−10. On average, more than 98% of them mapped on the *H. pylori* G27 reference genome. The ChIP-seq data quality was evaluated by using ENCODE quality metrics^1^ and the values obtained are provided in **Supplementary Table S2**. To perform peak calling, the Homer (v4.7.2) (Heinz et al., 2010) algorithm was used with default parameters. Briefly, the Homer algorithm finds non-random clusters of reads by looking at the tested sample alone, then each peak is required to have: (1) 4-fold more normalized reads in the sample experiment than in the background control and a cumulative Poisson *p*-value of 0.0001 to assess the chance that the differences in reads counts between sample and background are statistically significant; (2) read density 4-fold greater than in the surrounding 10 kb region; (3) the ratio between the number of unique positions containing reads in the peak and the expected number of unique positions given the total number of reads in the peak lower than 2. Only the peaks identified in both biological replicates and having overlapping genomic coordinates were considered significantly reliable and included in the final peak list. The peaks having their center within −100/ + 30 bp from a transcription start site (TSS) were defined as promotorial, while the remaining peaks were divided in intragenic, when their center was mapping within a predicted coding region, or intergenic, when it was mapping outside from annotated regions (see **Supplementary Table S1** and RNA-seq analysis paragraph for details). TSS were identified first by blasting 50 bp upstream of each of the transcription initiation sites reported by Sharma et al. (2010) in the HP26695 genome on the G27 genome and then by the positioning of our RNA-seq signals. Raw data are publicly available in Sequence Reads Archive under accession number BioProject PRJNA421261.

### DNA Techniques

DNA manipulations were performed as described by Sambrook et al. (1989). All restriction and modification enzymes were used according to the manufacturers’ instructions (New England Biolabs). Preparations of plasmid DNA were carried out with NucleoBond Xtra Midi plasmid purification kit (Macherey-Nagel).

### Overexpression and Purification of Recombinant HspR Protein

His_6_-tagged recombinant HspR protein was overexpressed in *E. coli* BL21 (DE3) cells and affinity purified as previously described (Spohn and Scarlato, 1999; Roncarati et al., 2007). The purified His-HspR protein was dialyzed against two changes of 1X footprinting buffer (10 mM Tris-HCl, pH 8.0; 50 mM NaCl; 10 mM KCl; 5 mM MgCl_2_; 0.1 mM DTT; 0.01% NP40) avoiding any trace of glycerol, prior to the DNA binding experiment, and stored at −80°C. Protein concentration was determined by Bradford colorimetric assay (BioRad) and purity assayed by SDS-PAGE.

### Construction of DNA Probes for *in vitro* DNA-Binding Assays

Genomic regions of *H. pylori* G27 encompassing HspR binding sites on *hrcA*, *groES*, *cbpA* promoters and *speA* coding region were PCR amplified with specific primers (**Table 2**) and cloned into the pGEM-T-Easy plasmid (**Table 1**). The M1, M3, M4 and M5 P*cbp* mutant probes were generated by annealing complementary oligonucleotides to form a double stranded DNA fragment with compatible overhangs required to clone it in the pGEM-T-Easy plasmid previously digested with the appropriate restriction enzymes. The M2 and M1+2 P*cbp* mutant probes were generated through site-directed mutagenesis using the plasmid pGEM-T-Easy, harboring the P*cbp* wild type sequence, as DNA template and primers listed in **Table 2**.

**Table 2:** Oligonucleotides used in this study.

Oligonucleotides	Nucleotide sequences (5’ to 3’)^a^	Restriction recognition site
RBS GroF	TCTTCAAAAAGGTTTGTTAATGACGC	None
RBS GroR	AGCACATTTTTAGGGATAAGTCAAGC	None
RBS hrc F	CGATTTTTCTTTAAAGTTTAGTCTGTATCAC	None
Phrc F	ATATGGATCCTACGTCAAGCAAGCGATAACTTTAC	None
HBS CbpF	AATTCCTTTTAATTGCACTGAAACGGG	None
HBS CbpR	GGTATAAACTCTTGCTCATGAATCACC	None
HBS speAF	CCACGAAGCCCTTGTTTTTGC	None
HBS speAR	CGCTAAATTCCGTAGGGTGC	None
Mut1 F	GATCCAAAATAGTTTTATTAGAATACTATCATAAATCAGGTACCTTAGTCAATCAAGTTTATTGATAATGTTTAGTGGTAATTGAGATTTG	None
Mut1 R	GTTTTATCAAAATAATCTTATGATAGTATTTAGTCCATGGAATCAGTTAGTTCAAATAACTATTACAAATCACCATTAACTCTAAACTTAA	EcoRI
Mut2 F	AGTCGACAGTTTATTGATAATGTTTAG	SalI
Mut2 R	CTAACACTAAAGATTTATGATAGTATTC	None
MUTD2R	CTAAGGTACCTGATTTATGATAGTATTC	KpnI
Mut3 F	GATCCAAAATAGTTTTATTAGAATACTATCATAAATCTTTAGTGGAGCTCAATCAAGTTTATTGATAATGTTTAGTGGTAATTGAGATTTG	None
Mut3 R	GTTTTATCAAAATAATCTTATGATAGTATTTAGAAATCACCTCGAGTTAGTTCAAATAACTATTACAAATCACCATTAACTCTAAACTTAA	None
Mut4 F	GATCCAAAATAGTTTTATTAGAATACAGGTACCAATCTTTAGTGTTAGTCAATCAAGTTTATTGATAATGTTTAGTGGTAATTGAGATTTG	None
Mut4 R	GTTTTATCAAAATAATCTTATGTCCATGGTTAGAAATCACAATCAGTTAGTTCAAATAACTATTACAAATCACCATTAACTCTAAACTTAA	None
Mut5 F	GATCCAAAATAGTTTTATTAGAATACTATCATAAATCTTTAGTGTTAGTCAATCAAGTTTTAGTCGACTGTTTAGTGGTAATTGAGATTTG	None
Mut5 R	GTTTTATCAAAATAATCTTATGATAGTATTTAGAAATCACAATCAGTTAGTTCAAAATCAGCTGACAAATCACCATTAACTCTAAACTTAA	None
LuxF	ATATGGATCCCAGGCTTGGAGGATACGTATGAC	BamHI
LuxR	ATATGGATCCGGCATTCGGTAATATATGCGC	BamHI
LuxRTF	ATCATCCGATAACGCGCTCTT	None
LuxRTR	ACCGCCCAATTAATCGCATC	None
cbpEco	ATATGAATTCAATTCCTTTTAATTGCACTGAAACGGG	EcoRI
cbpBam	ATATGGATCCGGTATAAACTCTTGCTCATGAATCACC	BamHI
cbpRTRev	TTTAGCTAGGCAATACCACCCGGA	None
hspRC	ATATATCTCGAGTTTTTTAAATAAAATCAGTTCATA	XhoI
cys-F	CGTTTTAGGGACTTTGGGAGG	None
vacA-R	GCTGGTTTTATGCTCTAAACTGG	None
ppk RT-F	CGCGCCTTTCTAAATTTCTGGGCA	None
ppk RT-R	CCCAAGTCAAAGGCTTGAGCGAAA	None

*^a^Nucleotides added to reconstitute the indicated restriction recognition sites are underlined.*

### Hydroxyl-Radical Footprinting Assay

Probe DNA fragments obtained by digestion with the appropriate restriction enzymes were 5′ end-labeled with [γ^32^ P]-ATP and T4 polynucleotide kinase and gel purified. Hydroxyl-radical footprinting experiments were performed as previously described (Pelliciari et al., 2017) with some modifications. Approximately 20 fmol of labeled probes were incubated with increasing concentration of HspR protein in hydroxyl-radical footprinting buffer (10 mM Tris-HCl, pH 8.0; 50 mM NaCl; 10 mM KCl; 5 mM MgCl_2_; 0.1 mM DTT; 0.01% NP40) for 15 min at room temperature, including 200 ng of sonicated salmon sperm DNA as a non-specific competitor in a final volume of 30 μl. Partial digestions of the labeled probes were achieved using 2 μl each of the following solutions: 125 mM Fe (NH_4_)_2_(SO_4_)_2_ 250 mM EDTA, 1% H_2_O_2_ and 100 mM DTT. After 2 min incubation, the reaction was quenched by the addition of 25 μl of OH Stop Buffer (4% glycerol; 600 mM NaOAc, pH 5.2; 100 ng/μl sonicated salmon sperm DNA). Samples were phenol/chloroform extracted, ethanol precipitated and resuspended in 12 μl of Formamide Loading Buffer. Next, samples were denatured at 100°C for 5 min, separated on a 8M urea-8.4% polyacrylamide sequencing gel in TBE buffer and autoradiographed.

### Generation of *H. pylori* P*cbp-lux* Reporter Strains

The wild type P*cbp* and mutated P*cbp* M1 + 2 promoter regions were PCR amplified with specific primers listed in **Table 2**, using as template the *H. pylori* G27 genomic DNA and the plasmid pGEM-T-Easy-HBScbp M1 + 2 (**Table 1**), respectively. Then, the generated DNA fragments were digested with appropriate restriction enzymes and cloned into the pVAC plasmid (**Table 1**). The *luxC* gene was PCR amplified from the pVCC vector (**Table 1**) and cloned downstream the P*cbp* promoters into the pVAC vector. These plasmids were used to transform the *H. pylori* G27 wild type acceptor strain in the *vacA* locus. The chloramphenicol-selected mutant strains were expanded and the correct insertion was confirmed by PCR using oligonucleotides pair cys-F/vacA-R as primers (**Table 2**). The *H. pylori* P*cbp*-*lux, hspR::Km* reporter strains (**Table 1**) were generated transforming the *H. pylori vacA::*P*cbp* wt*-lux* and the *H. pylori vacA::*P*cbp*M1 + 2*-lux* acceptor strains (**Table 1**) with the plasmid pGEM3-*hspR::Km* (**Table 1**). The kanamycin-selected mutant strains were expanded and the correct insertion was confirmed by PCR using oligonucleotides pair cbpRTRev/hspRC as primers (**Table 2**).

### qRT-PCR Analysis

Synthesis of cDNA and qRT-PCR analysis were carried out as previously described (Pelliciari et al., 2015). Briefly, for cDNA synthesis 1 μg of DNA-free RNA was incubated with 50 ng of random hexamers (Invitrogen), dNTPs mix (1 mM each), 5 U of AMV-Reverse Transcriptase (Promega), and incubated for 1 h at 37°C. For qRT-PCR analyses, 2 μl of diluted (1:10) cDNA samples were mixed with 5 μl of 2X Power Up SYBR Green master mix (ThermoFisher Scientific) and specific oligonucleotides for the genes of interest (**Table 2**) at 400 nM concentration in a final volume of 10 μl. qRT-PCR experiments were performed using the following cycling protocol: 95°C for 2 min, then 40 cycles consisting of a denaturation step for 5 s at 95°C followed by 30 s at 60°C (annealing and extension steps). Data were analyzed using the ΔΔCt method, using the housekeeping *ppk* gene, known to be constitutively expressed, as internal reference, using oligonucleotides ppk RT-F and ppk RT-R as primers (**Table 2**) (Muller et al., 2011; Agriesti et al., 2014).

## Results

### RNA-seq Analysis Identifies HspR-Dependent Heat-Shock Gene Transcripts

To define the HspR contribution to the heat-shock response, we performed a strand-specific whole transcriptome analysis of the wild type *H. pylori* G27 strain and of *ΔhspR* mutant both grown to the exponential growth phase at 37°C and of the wild type strain subjected to 30 min heat-shock at 42°C (**Supplementary Table S2**, Materials and Methods).

**FIGURE 1:**
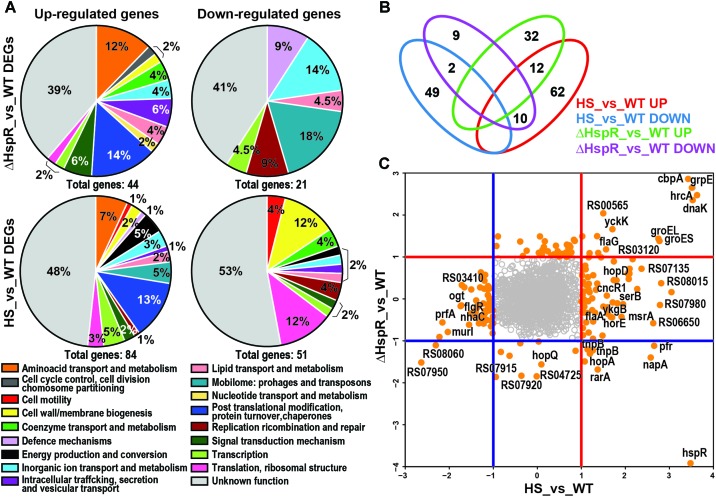
Role of HspR in heat-shock response. **(A)** Pie charts showing COGs functional annotation of the differentially expressed genes outlined in the *Δ*HspR_vs_WT and HS_vs_WT (Heat-Shock vs. Wilde Type) comparisons, respectively, subdivided into up-regulated (left) and down-regulated (right) groups. The abundance of each category is indicated as a percentage as well as the total number of genes included in each group. **(B)** Venn diagram showing the number of genes in common or peculiar in the four previously described gene groups, red numbers highlight commonly regulated genes. **(C)** log2FC plot showing for each gene HS_vs_WT value on x-axis and HspR_vs_WT value on y-axis. DEGs (log2FC > |1| and *p*adj < 0.01 are represented as orange filled circles; empty gray circles correspond to non-differentially expressed genes. Blue and red lines indicate log2FC < −1 and log2FC > 1 thresholds, respectively, subdividing coherently and incoherently regulated genes.

We defined the role of HspR in standard growth conditions by comparing the transcriptome of the *ΔhspR* mutant to that of the *H. pylori* wild type strain, both grown at 37°C (*ΔhspR*_vs_WT). This analysis showed a total of 65 deregulated genes (log2FC > |1| *p*adj < 0.01) upon *hspR* gene deletion. Of these, 21 were down-regulated and 44 were up-regulated genes (**Figure 1A** and **Supplementary Table S3**, sheet B). As expected, among the up-regulated genes we found *groES, groEL, grpE, dnaK*, and *cbpA* (Spohn and Scarlato, 1999), which strongly contributed to “Post-translational modification, protein turnover, chaperones” functional enrichment among (*p*adj = 0.01) *ΔhspR* de-repressed genes (**Figures 1A,B**), and *hrcA* transcriptional regulator (**Figure 1B**). As expected, because of the *hspR* genomic deletion, this gene appeared as down-regulated. The *rarA* gene, mapping downstream of the *hspR* gene, possibly due a polar effect of the deletion mutant also appeared as a down-regulated gene. Several transposase coding genes, producing the “Mobilome: prophages, transposons” category enrichment (*p*adj = 0.00009), and several genes involved in the “Inorganic ion transport and metabolism” were also down-regulated.

Upon heat-shock at 42°C the transcriptome of the *H. pylori* wild type G27 strain showed a total of 134 differentially expressed genes (log2FC > 1 *p*adj < 0.01) when compared to the wild type sample not subjected to heat-shock (HS_vs_WT). Of these genes, 83 were up-regulated and 51 were down-regulated (**Supplementary Table S3**, sheet A). Functional annotation and enrichment analysis (see Materials and Methods) revealed that up-regulated genes were enriched in “Post-translational modification, protein turnover, chaperones” (*p*adj = 0.0007) and “Mobilome: prophages, transposons” class (*p*adj = 0.008; **Figure 1A**). Several of the 51 down-regulated genes were also annotated to “Cell wall/membrane/envelope biogenesis” and “Translation, ribosomal structure and biogenesis” categories (**Figure 1A**) without statistical significance.

Comparing the transcriptome of the heat-shock response (134 deregulated genes) to that of the *hspR* deletion mutant (65 deregulated genes) we identified 25 genes that were deregulated in both datasets (**Figures 1B,C**, red numbers). Among these genes, 10 were oppositely regulated and 14 were similarly regulated: 2 down-regulated and 12 up-regulated (**Figure 1B**, bottom left and right quadrants of the graph and **Figure 1C**). The similarly up-regulated genes included all the genes of the operons already known to be directly repressed by HspR and induced by heat-shock (Spohn and Scarlato, 1999). In addition, we found genes coding for amino acid transporters *yckK* and *yckJ*, flagellar protein *flaG*, a polyisoprenoid-binding protein and two genes coding for hypothetical proteins, which could be new targets under HspR direct control (**Figures 1B,C**). The remaining eleven genes were induced by heat-shock and repressed in the *hspR* mutant strain, thus showing opposite behavior. Considering that HspR acts as a repressor factor, these genes are probably indirectly controlled by HspR.

Overall, this analysis highlights that HspR could be involved in the control of maximum 18% (25 out of 134) of the genes whose transcription is heat responsive and that it represses the transcription of only 14 genes.

### Genome-Wide *in vivo* Identification of HspR Binding Sites Through ChIP-seq

**FIGURE 2:**
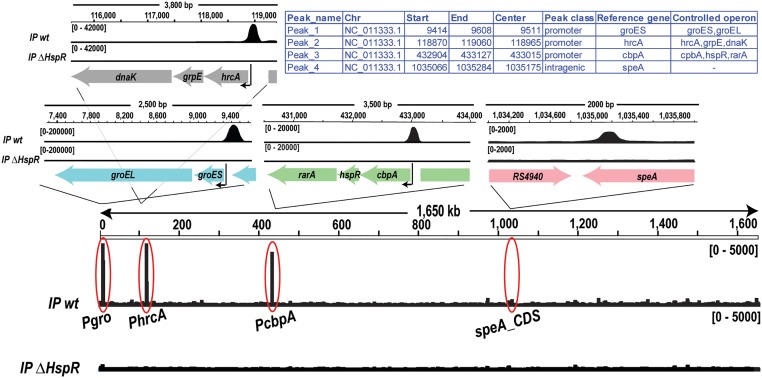
Genome-wide *in vivo* identification of the HspR binding sites. Genome-wide signals of HspR bindings in the wild type genotype (IP wt, upper track) and in *hspR* deletion mutant used as negative control (IP ΔHspR, lower track). The regions corresponding to the four HspR binding sites revealed by the analysis are magnified in the upper panels showing each peak in its genomic context including annotated genes (thick arrows) and the transcriptional start site (black thin arrow), when present.

To identify the genomic regions bound *in vivo* by HspR, therefore, the genes directly controlled by HspR binding, we performed a Chromatin Immunoprecipitation assays followed by deep sequencing (ChIP-seq) in *H. pylori* G27 wild type and *ΔhspR* strains (**Figure 2**). To identify HspR bound regions (peaks), ChIP-seq signals obtained from each wild type sample (IP wt) were compared to those resulting from the pool of *ΔhspR* mutant samples (IP *ΔhspR*) (see Material and Methods for details). Only the significant peaks in both replicates were considered in the final peak list. Surprisingly, this analysis identified only four reproducible HspR binding regions (**Figure 2**), which were annotated with respect to the latest genome annotation of the *H. pylori* G27 strain (GCF_000021165.1). According to this annotation, three peaks were classified as promotorial and confirmed HspR direct control for 8 genes of the *groES-groEL*, *hrcA-grpE-dnaK* and *cbpA-hspR-rarA* operons. The remaining peak, mapping inside the coding region of *speA* gene, was classified as intragenic. Thus, these data suggest that the HspR direct regulon is very restricted and limited to the three multicistronic heat-shock operons. Moreover, through ChIP-seq analysis we confirmed *in vivo* also the existence of at least one intracistronic binding site, apparently not associated to transcriptional regulation.

### HspR Binds Extended DNA Regions and Adopts a Peculiar Binding Architecture

**FIGURE 3:**
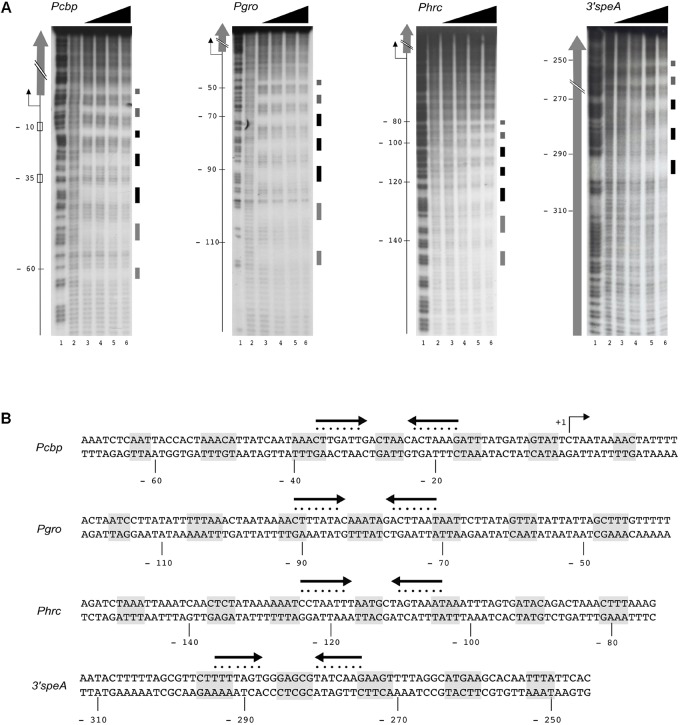
HspR binds extended DNA regions. **(A)** Hydroxyl-radical footprint experiments on HspR positive target genes identified by ChIP-seq analysis. From left to right, specific DNA probes for P*cbp*, P*gro*, P*hrc*, and *speA* fragments were radioactively end-labeled on the coding strands, and incubated with increasing amounts of purified HspR protein prior hydroxyl-radical digestion. P*cbp*, P*gro*, P*hrc* labeled probes were mixed with 0; 47.5; 95; 190; 380 nM HspR (lanes 2–6); while *speA* probe was mixed with 0; 60.7; 121.5; 243; 486 nM HspR (lanes 2–6). Purified DNA fragments were separated on a polyacrylamide denaturing gel along with a G + A sequence reaction ladder as reference marker (lane 1 in all the panels). On the right of each panel, black and gray boxes represent strong and weak HspR protected regions, respectively. On the left, the –10 and –35 regions and the transcriptional start site (+1, bent arrow) are indicated and the open reading frames are depicted with vertical gray arrows. The relative position of the HspR binding site on the promoter regions of P*cbp*, P*gro*, P*hrc* is reported with respect to the transcriptional start site, while those mapped on the coding sequence of *speA* gene are reported with respect to the translational stop codon. **(B)** Nucleotide sequences of HspR binding sites on the promoter regions of the three heat-shock operons (P*cbp*, P*gro*, P*hrc*) and on the 3′ coding region of the *speA* gene. The HspR protected regions identified in **(A)** are shaded in gray, while the inverted repeat sequences similar to the HAIR consensus motif are depicted as converging black arrows and each nucleotide of the motif is marked with a dot. Nucleotide positions with respect to the transcription initiation sites are reported on the non-coding strand.

Previous DNase I footprinting assays showed that *H. pylori* HspR binds directly to extended DNA regions of the promoters of the three heat-shock operons (Spohn and Scarlato, 1999; Roncarati et al., 2007). In particular, binding of HspR to its operators resulted in protection of large DNA regions of 70–80 bp with six bands of enhanced DNase I sensitivity at both adjacent and internal sites. In order to further characterize HspR-DNA interactions and to get more detailed information on the HspR DNA-binding architecture, we carried out hydroxyl-radical footprinting experiments on the genomic targets found in ChIP-seq assay. **Figure 3A** shows the results of hydroxyl-radical footprintings performed with increasing concentrations of recombinant purified HspR on the promoter region of the three heat-shock operons (P*cbp*, P*gro*, P*hrc*) and on the 3′ region of the *speA* coding sequence. According to previous observations (Spohn and Scarlato, 1999; Delany et al., 2002c; Roncarati et al., 2007) the protected regions on the P*cbp*, P*gro* and P*hrc* promoters map, respectively, from position −63 to +10, from −117 to −44 and from −150 to −82 with respect to the transcriptional start site. Furthermore, the HspR binding site located in the coding region of *speA* gene spans from nucleotide position −298 to −250 with respect to the translational stop codon. Intriguingly, HspR binding on these targets results in a peculiar periodic pattern of short protected regions from radical digestion, which appears to be slightly different between the promotorial and intragenic binding sites. Indeed, HspR binding on P*cbp*, P*gro* and P*hrc* promoter regions results in 7 short protected DNA tracts separated by non-protected regions of 7/8 nucleotides, while the binding site located inside the *speA* coding sequence appears at higher protein concentrations and is characterized by 5 short protected regions instead of 7. Data obtained from DNA-binding assays are schematized in **Figure 3B**, which reports the nucleotide sequence of the HspR binding sites (operator) on the P*cbp*, P*gro*, P*hrc* promoters and on the *speA* coding region. Notably, in all the operators we identify one inverted repeat (represented by converging arrows in **Figure 3B**) similar to the HAIR motif proposed as a consensus sequence for the HspR protein of *S.* *coelicolor* (CTTGAGT-N7-ACTCAAG) (Grandvalet et al., 1999). It is worth noting that in the operators of the three heat-shock operons’ promoters the inverted repeat is located in a central position of the HspR binding sites, suggesting that it could play an important role in nucleating HspR binding to DNA. Therefore, the heat-shock transcriptional repressor HspR binds, with a peculiar DNA-binding pattern of short stretches of protected regions, spanning over about 70-80 bp of DNA harboring a conserved inverted repeat similar to the HAIR consensus sequence of *Streptomyces* spp.

### The Central HAIR-Like Motif Is Essential for HspR Binding to DNA

**FIGURE 4:**
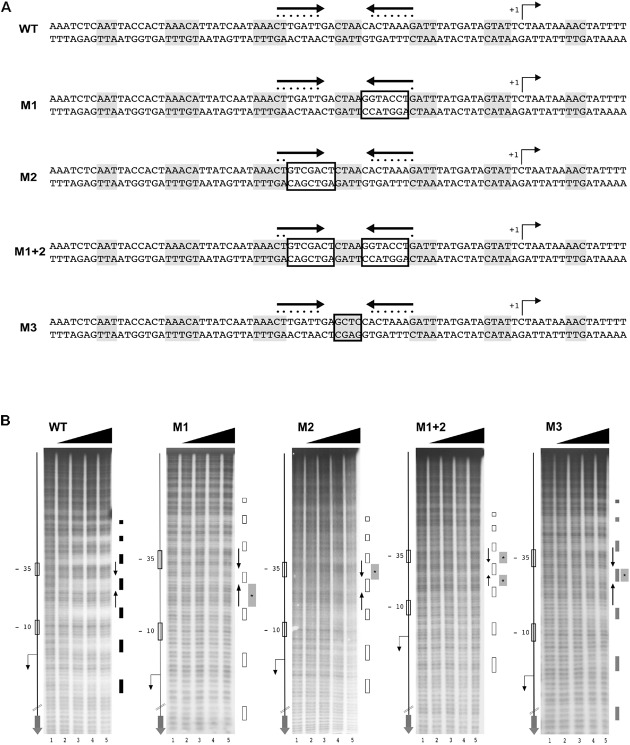
The HAIR-like motif is essential for HspR binding to DNA. **(A)** Schematic representation of wild type and four different P*cbp* mutant probes, in which one (M1 and M2) or both arms (M1 + 2) of the inverted repeat and the protected region between these sequences (M3) have been mutagenized by base substitution. HspR protected regions on P*cbp* are shaded in gray and the inverted repeat sequences (HAIR-like motif) is represented by converging black arrows and their nucleotides marked with dots. In each DNA probe, mutagenized nucleotides are boxed. **(B)** From left to right, hydroxyl-radical footprint experiments on wild type and the indicated mutants of the P*cbp* promoter region. Symbols are as described in the legend to **Figure 3A**. Wild type (WT) and indicated mutants DNA probes were incubated with increasing amounts of purified HspR protein and submitted to hydroxyl-radical digestion (see legend to **Figure 3**). Black, gray, and empty boxes to the right of each panel denote strong, weak, and loss of protection by HspR, respectively. Black converging arrows to the right of each panel mark the positions of the HAIR-like inverted repeat sequences, while gray boxes with an internal asterisk indicate the mutagenized regions.

To ascertain the functional importance of the HAIR-like sequence elements in HspR binding to DNA, we decided to introduce bases substitutions in the inverted repeat and monitor their effects on HspR DNA-binding through hydroxyl-radical footprinting assays. A schematic representation of the wild type and mutant sequences of the HspR binding site on the P*cbp* region is reported in **Figure 4A**. As shown in **Figure 4B**, mutation of one or both arms (M1, M2, or M1 + 2) of the HAIR-like sequence completely abolished HspR binding to the DNA in the concentration range tested. In particular, partial (M1 and M2) or total substitution (M1 + 2) of the HAIR-like sequence prevented HspR binding to both, the mutagenized sequence (central portion of the probe) and the flanking upstream and downstream regions of the HAIR-like motif. It is worth mentioning that regions protected in hydroxyl-radical footprinting experiments reflect limited accessibility of radical ions to the DNA minor groove and, for this reason, these protected regions do not necessarily represent the portions of the probe directly contacted by the HspR protein, but regions surrounding short stretches of contacted nucleotides. Considering that HspR footprint regions surround the conserved HAIR-like element (**Figure 4**), our data suggest that HspR could interact with the HAIR-like element in the DNA major groove narrowing the adjacent minor grooves, which results in the protection observed *in vitro* by hydroxyl-radical footprinting. Furthermore, mutation of the non-conserved sequence in between the two inverted repeats (probe M3 in **Figure 4A**) showed a barely detectable protection of HspR only upon addition of high amount of the protein to the reaction (**Figure 4B**, lane 6 of probe M3). This is likely due to a significant loss of protein affinity to the DNA, suggesting that, besides the HAIR-like motif, also this non-conserved DNA element is important for HspR binding. These data show for the first time that in *H. pylori* the HAIR-like sequence is an essential DNA element for the specific binding of HspR to the P*cbp* promoter region. Likely, HspR binding to the operator is driven by a specific recognition of determinants within the HAIR-like sequence, including the non-conserved spacer between the inverted repeat.

**FIGURE 5:**
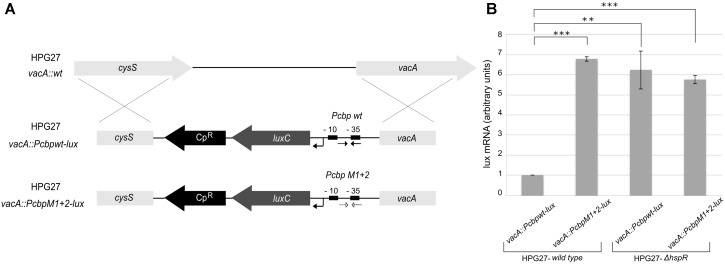
*In vivo H. pylori* transcripts levels from the P*cbp* promoter harboring WT or mutant HAIR-like sequences. **(A)** Schematic representation of P*cbpwt*-*lux* and P*cbpM1 + 2*-*lux* reporter constructs obtained transforming the *H. pylori* G27 wild type acceptor strain by double homologous recombination in the *vacA* locus, and selected by chloramphenicol resistance (Cp^R^). The wild type (P*cbpwt*-*lux*) or HAIR-like mutant (P*cbpM1 + 2*-*lux*) P*cbp* promoter is inserted upstream of a *luxC* reporter gene. The HAIR inverted repeat sequences are indicated by converging black arrows in the P*cbp* WT promoter (P*cbpwt*-*lux*) and by converging dotted arrows in the P*cbp* HAIR-like mutant promoter (P*cbpM1 + 2*-*lux*). In each reporter construct, the –10 and –35 regions are depicted as black boxes and the transcriptional start site as a bent arrow. **(B)** Transcript levels of P*cbp* wild type and P*cbp* HAIR-like mutant promoters fused with *lux* reporter gene were assayed by qRT-PCR in the wild type and *hspR* deletion mutant strains using specific oligonucleotides for the *luxC* gene (LuxRTF/R). Mean values from three independent biological samples are reported in the graph, with error bars indicating standard deviation and asterisks marking statistical significance calculated by a Student’s *t*-test (^∗∗∗^*p*-value < 0.001; ^∗∗^*p*-value < 0.01).

To characterize *in vivo* the functional significance of the central HAIR-like motif, we generated a reporter construct in which the wild type or HAIR-like mutant P*cbp* promoter (P*cbp*M1 + 2) was fused upstream of a 5′ fragment of the *luxCDABE* reporter cassette and, upon integration in *H. pylori* chromosome, transcript level was assayed (through RT–qPCR with *luxC* specific primers) during exponential growth (**Figure 5**). When the central HAIR-like inverted repeat was mutated in the G27 wild type strain, a consistent increase in the amount of transcript from the P*cbp*M1 + 2 promoter with respect to the wild type P*cbp* promoter was observed (**Figure 5B**). The about 6.5-fold increase of transcript from the P*cbp*M1 + 2 promoter is in line with the previously observed de-repression of the P*cbp* promoter in the *H. pylori* *hspR*-mutant strain (Spohn et al., 2004). Accordingly, in a *hspR* null background a similar high amount of transcripts from the P*cbpwt* and the P*cbp*M1 + 2 promoters was observed (**Figure 5B**). These *in vivo* data are consistent with the *in vitro* observations, indicating that the central HAIR-like motif drives specific binding of HspR to its operator on P*cbp* with concomitant promoter transcriptional repression.

### Non-conserved DNA Regions, Surrounding the HAIR-Like Motif, Are Necessary for HspR to Fully Occupy Its Extended DNA Binding Site

**FIGURE 6:**
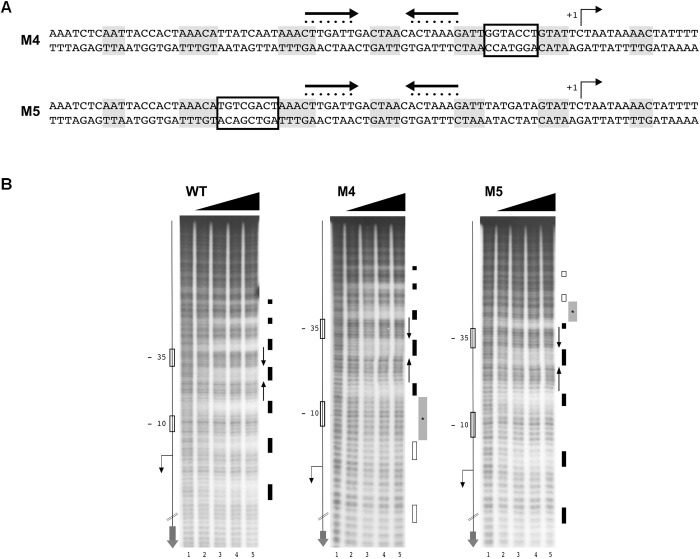
Non-conserved regions surrounding the HAIR-motif are required for HspR binding. **(A)** Schematic representation of WT and two P*cbp* mutant probes, in which the spacer regions highlighted with empty boxes were mutagenized by base substitution. Symbols are as in the legend to **Figure 3**. **(B)** From left to right, hydroxyl-radical footprint experiments on WT and mutant probes described in **(A)** (M4 and M5) with increasing amounts of purified HspR protein detailed in the legend to **Figure 3**.

In order to understand if DNA sequences flanking the HAIR-like motif are important for HspR-DNA recognition and binding, we designed two mutant probes of the P*cbp* promoter and assayed for HspR binding by hydroxyl-radical footprintings. Mutations were introduced in the non-conserved spacer region between two 4-bp protected tracts on both sides of the inverted repeat (**Figure 6A**, M4 and M5). Surprisingly, as shown in **Figure 6B**, the addition of increasing amounts of HspR to the mutant probes led to the disappearance of regions of protection (marked in light gray) only on the side of the mutated region, while it was unaffected on both the inverted repeat of the HAIR-like sequence and on the opposite side of the mutation (**Figure 6B**, M4 and M5). In conclusion, these data support the pivotal role of the HAIR-like motif for HspR-DNA binding in *H. pylori* and demonstrate that also other non-conserved DNA regions surrounding the HAIR-like motif are important elements that allow HspR to completely occupy its extended binding sites.

## Discussion

The heat-shock response is a universal mechanism of cellular protection against sudden adverse environmental growth conditions and has been observed in every bacterial species investigated (Roncarati and Scarlato, 2017). It consists of a set of well-coordinated responses and processes, mostly involving the strictly regulated expression of various heat-shock proteins (HSPs) and chaperones. In *H. pylori* their expression level is tightly modulated by the concerted action of two transcriptional repressors, HrcA and HspR, with this latter acting as the master regulator of this circuit (Danielli and Scarlato, 2010). In the present study, we examined the heat-shock regulon in *H. pylori* and discriminated between direct and indirect transcriptional response mediated by HspR. As shown by transcriptome analyses, heat-shock treatment triggered changes in the transcript levels of 135 genes. Of these, 83 genes appeared up-regulated and 51 genes appeared down regulated (**Figure 1A**). Accordingly, the heat-shock operons *groES-groEL*, *hrcA-grpE-dnaK* and *cbpA-hspR-rarA*, coding for the major chaperones and heat-shock proteins of *H. pylori* were clearly up-regulated. With the exception of the *hspR* and *rarA* genes, these operons, known to be directly repressed by HspR (Spohn and Scarlato, 1999; Roncarati et al., 2007), were coherently up-regulated also in the *ΔhspR* mutant strain. On the other hand, HspR seems to affect in a positive or negative manner the transcription of other 59 genes involved in diverse cellular processes and not strictly associated to heat-shock (**Figure 1A**). In contrast, ChIP-seq analysis showed only four already known *in vivo* HspR genomic binding sites, three of which are associated to the promoter regions of the heat-shock operons and one mapping within the coding sequence of the *speA* gene (**Figure 2** and Spohn and Scarlato, 1999; Delany et al., 2002c; Roncarati et al., 2007). This apparent discrepancy between the RNA-seq and ChIP-seq results is in agreement with other HspR studies conducted in *S. coelicolor* and *Mycobacterium tuberculosis* (Stewart et al., 2002; Bucca et al., 2003). In these bacteria, HspR alone or in combination with other transcriptional regulators controls transcription of a limited number of genes coding for chaperones and heat-shock proteins. In *H. pylori*, *hspR* deletion affects the transcript abundance of a high number of genes not directly controlled by HspR. Likely, regulation by HspR can also be exerted in an indirect manner through a still unknown molecular mechanism. Recalling the presence of the *hrcA* gene among the direct targets of HspR, it is tempting to speculate that many of the genes deregulated by the *hspR* deletion and lacking a HspR binding site arise from altered levels of the HrcA regulator in the *ΔhspR* mutant. Furthermore, the possibility that up- and/or down-regulated genes in the *ΔhspR* mutant strain might arise from the enhanced synthesis of one or more members of the HspR regulon cannot be ruled out and remains to be elucidated.

Although several chaperones and heat-shock proteins of *H. pylori* are induced by a temperature upshift (**Supplementary Table S3**), surprisingly, the transcription of genes coding for stress related proteases seems to be unaffected by the heat challenge. Furthermore, while in some other bacteria, like for example *Campylobacter jejuni* and *S. coelicolor*, several protease-encoding genes have been shown to belong to the HspR regulon (Bucca et al., 2003; Holmes et al., 2010), we demonstrated that in *H. pylori*, these genes are not controlled by the HspR repressor. Considering that the amount of proteases is expected to increase in response to different stress insults encountered by the pathogen, an interesting hypothesis considers the existence of post-transcriptional or post-translational control strategies, which would provide enhanced levels of these crucial players during adverse environmental growth conditions.

**FIGURE 7:**
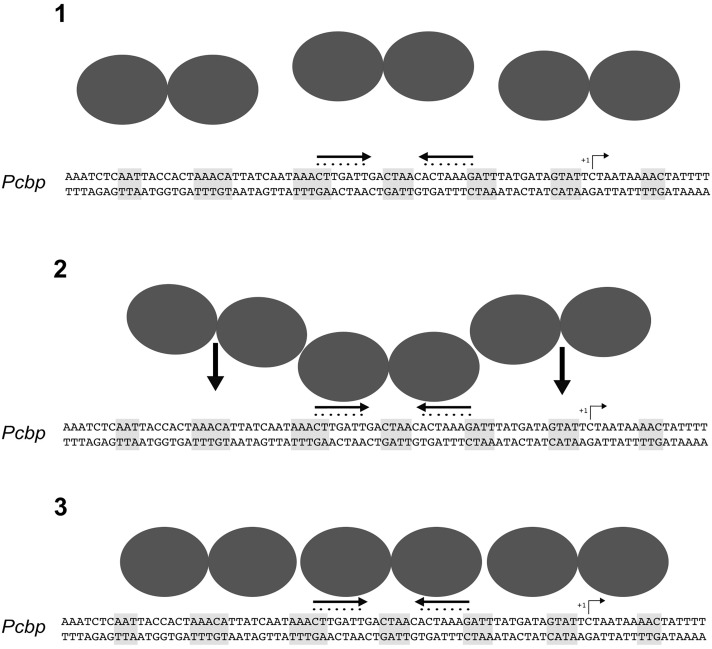
Model of the molecular mechanism for HspR-DNA binding. In solution, HspR dimerizes (1) and binds with high affinity to the DNA region containing the HAIR consensus sequence (2). This binding could act as a nucleation center driving cooperative binding of two HspR dimers on the flanking regions (3), likely recognizing still unknown sequence determinants.

Previous DNase I footprinting assays of HspR showed protection of large DNA regions of about 70 bp upstream of the promoters controlling transcription of three heat-shock operons (Spohn and Scarlato, 1999; Roncarati et al., 2007). To deepen our understanding of the molecular mechanisms controlling HspR-DNA interactions, we set up high-resolution hydroxyl-radical footprinting assays on its target gene probes. Results showed that HspR binds extended DNA regions with a peculiar short periodic pattern, which appears to be slightly different between promoter and intragenic regions (**Figure 3A**). Binding of HspR to promoter regions shows 7 short protected DNA tracts spaced by non-protected regions of 7/8 nucleotides, while binding within the *speA* coding sequence appears at higher protein concentrations and shows only 5 short protected regions. This different binding pattern could be related to the fact that the latter binding site on the coding sequence of *speA* gene appears to be not associated to transcriptional regulation. In fact, RNA-seq analysis revealed no changes in the level of *speA* transcript in the *hspR* mutant strain, nor of neighboring genes. This is not surprising, as the advent of the “omics” era highlighted binding of regulatory proteins to a number of binding sites not associated to regulation, such as the *H. pylori* Fur repressor (Danielli et al., 2006; Vannini et al., 2017), the *E. coli* CRP activator, and the RNA polymerase enzyme (Grainger et al., 2005). However, all four *H. pylori* HspR binding sites show an inverted repeat with similarities to the HAIR consensus sequence of *Streptomyces* spp. (Grandvalet et al., 1999) (**Figure 3B**). These HAIR-like motifs map in the central position of the HspR binding sites on the three heat-shock operons’ promoters and appear to be an essential DNA element for specificity of protein binding. In fact, mutation of one or both arms of this inverted repeat completely abolished the HspR binding to the operator (**Figure 4**) and prevented *in vivo* HspR-dependent repression of P*cbp* (**Figure 5**), demonstrating for the first time that the HAIR-like sequence is functionally important also in *H. pylori*. The DNA-binding mechanism of HspR on its target operators is unique among *H. pylori* transcriptional regulators characterized so far. Well-studied *H. pylori* regulatory proteins, such as HP1043, HrcA, NikR and Fur appear to recognize conserved sequence motifs as dimers and protect limited DNA regions (Roncarati et al., 2014, 2016; Pelliciari et al., 2017; Vannini et al., 2017). In the case of Fur repressor, however, it has been shown that several Fur-regulated promoters harbor multiple Fur boxes and, upon Fur binding, large regions of the promoter result occupied by this metal-dependent repressor (Delany et al., 2002a; Roncarati et al., 2016). However, data presented in this work suggest a completely different DNA-binding mechanism for HspR. HspR-controlled promoters are characterized by a single, conserved inverted repeat (HAIR-like sequence) that drives DNA specific recognition. In contrast to other *H. pylori* regulators, HspR DNA binding extends over a large portion of the promoter DNA, resulting in the characteristic extended protection in *in vitro* footprinting assays (Roncarati et al., 2007). This peculiar behavior of *H. pylori* HspR appears to be different from the other HspR homologs that have been characterized at the molecular level. For example, in *S. coelicolor* HspR binds extended DNA regions harboring three inverted repeat sequences (IR1, IR2, IR3) in the promoter region of its DNA targets (Bucca et al., 1995), while in *H. pylori* HspR requires only one inverted repeat on the center of the binding site. Also, no additional conserved sequences similar to the HAIR-like motif have been detected. Therefore, we suppose that the molecular mechanism through which *H. pylori* HspR binds to such extended DNA regions is peculiar and differs from the one adopted by the *S. coelicolor* HspR. Moreover, site-directed mutagenesis of the P*cbp* promoter pointed out that additional non-conserved DNA regions located between (**Figure 4B**, M3) and flanking (**Figure 6B**, M4 and M5) the HAIR-like motif are important elements for DNA recognition and binding of HspR to the operator. Intriguingly, mutation of one of these elements impaired binding of HspR on the side of the mutation and showed no effects on the binding on the HAIR-like motif on the opposite side of the mutation (**Figure 6**). This finding is compatible with a cooperative mechanism of HspR binding. Possibly, a dimer of HspR recognizes and binds to the HAIR-like sequence and this in turn drives binding of additional dimers on both sides of the HAIR-like motif (**Figure 7**). Evidences that HspR could bind its DNA sequences as a dimer have been shown in previous studies, in which it was demonstrated that homologs of *H. pylori* HspR repressor protein could exists in a dynamic state between the dimeric and monomeric forms in solution (Parijat and Batra, 2015). Moreover, Spohn et al. (2004) provided evidences that *H. pylori* HspR is able to form high order oligomers. In conclusion, these results provide a more detailed comprehension of the interaction between HspR and its target DNA sequences and, at least for the P*cbp* promoter, let us to propose a cooperative DNA-binding mechanism of three HspR dimers on this operator as schematized in **Figure 7**. In this model, an HspR dimer binds to the central DNA region harboring the HAIR-like motif that acts as a nucleation center for protein–protein interactions driving cooperative binding of HspR homodimers to the specific and still unknown determinants of the DNA sequences surrounding the HAIR-like motif. Giving the low level of sequence conservation of the HAIR-like motifs across the HspR binding sites and the imperfect nature of the inverted repeats (i.e., low conservation of the two arms of the inverted repeats), it can also be speculated that HspR DNA binding could be driven by sequence-dependent local shape variations rather than by a base readout mechanism, or by a combination of these two mechanisms. In other words, the crucial role of the HAIR-like motifs for HspR binding could be explained by taking into account local DNA shape variations imposed by a peculiar succession of purine/pyrimidine, rather than considering only the unique chemical signatures of the individual DNA bases. This behavior is common among regulators of MerR family, to which the *H. pylori* HspR belongs to, employing indirect (shape) readout as part of their DNA binding mechanisms. These considerations could help also to explain our data on the importance of the central region of the HAIR-like motif and of the non-conserved regions on both sides of the HAIR-like motif (**Figure 4**, M3 and **Figure 6**, M4 and M5).

## Author Contributions

AD, CP, DR, and VS conceived and designed the experiments. SPE, EP, EF, TB, CP, and AV performed the experiments. SPE, EP, and SPU carried out the data analysis. DR and VS wrote the paper with contributors CP and EP. All the authors reviewed the manuscript.

## Conflict of Interest Statement

The authors declare that the research was conducted in the absence of any commercial or financial relationships that could be construed as a potential conflict of interest.

## Footnotes

1 https://code.google.com/archive/p/phantompeakqualtools/

## Supplementary Material

TABLE S1 Annotation of coding genes.

TABLE S2 Library preparation and sequencing performances.

TABLE S3 Transcriptome of the wild type and *ΔhspR* strains.
